# Supplementary material for: Modelling Skylarks (Alauda arvensis) to Predict Impacts of Changes in Land Management and Policy: Development and Testing of an Agent-Based Model
Source: PLoS One. 2013 Jun 6;8(6):e65803. doi: 10.1371/journal.pone.0065803 (PMC3675089; doi:10.1371/journal.pone.0065803)
Supplement: Supporting Information S4 — The skylark ODdox as a zipped archive. (ZIP) [file pone.0065803.s004.zip › Skylark_ODdox/classprobe__data-members.html]

ALMaSS Skylark ODdox: Member List


ALMaSS Skylark ODdox
 2.0


- Main Page
- Related Pages
- Classes
- Files

- Class List
- Class Index
- Class Hierarchy
- Class Members

probe\_data Member List

This is the complete list of members for probe\_data, including all inherited members.

|  |  |  |
| --- | --- | --- |
| CloseFile() | probe\_data |  |
| FileAppendOutput(int No, int time) | probe\_data |  |
| FileOutput(int No, int time, int ProbeNo) | probe\_data |  |
| m\_FileRecord | probe\_data |  |
| m\_MyFile | probe\_data | protected |
| m\_MyFileName | probe\_data | protected |
| m\_NoAreas | probe\_data |  |
| m\_NoEleTypes | probe\_data |  |
| m\_NoFarms | probe\_data |  |
| m\_NoVegTypes | probe\_data |  |
| m\_Rect | probe\_data |  |
| m\_RefEle | probe\_data |  |
| m\_RefFarms | probe\_data |  |
| m\_RefVeg | probe\_data |  |
| m\_ReportInterval | probe\_data |  |
| m\_TargetTypes | probe\_data |  |
| m\_Time | probe\_data | protected |
| OpenFile(char \*Nme) | probe\_data |  |
| OpenForAppendToFile() | probe\_data | inline |
| probe\_data() | probe\_data |  |
| SetFile(ofstream \*F) | probe\_data |  |
| ~probe\_data() | probe\_data |  |


- Generated on Thu Jan 10 2013 13:15:37 for ALMaSS Skylark ODdox by
   1.8.1.1
